# Sexually transmitted infections in the non-European Union and European Economic Area of the World Health Organization European Region 2021–2023

**DOI:** 10.1186/s12889-025-22630-6

**Published:** 2025-04-25

**Authors:** Machiko Otani, Jane Rowley, Viatcheslav Grankov, Giorgi Kuchukhidze, Stela Bivol, on behalf of WHO European Region non-EU/EEA STI Surveillance network, Adela Vasili, Jennifer Fernández Garcia, Hovhannes Hovhannisyan, Oleg Salimov, Hanna P. Muzychenka, Khatuna Zakhashvili, Ketevan Galdavadze, Nino Lomia Israel, Rivka Rich, Elvira Torobekova, Thomas Althaus, Alma Cicic, Milena Lopicic, Dragan Kochinski, Aurelia Popov, Claudio Muccioli, Danijela Simic, Jean-Luc Richard, Thibault Lovey, Sakina Shoeva, Taliha Karakök, Mehmet Balcı, Liudmila Polanska, Dilmurad Zhumanov

**Affiliations:** 1 World Health Organization Regional Office for Europe, Copenhagen, Denmark; 2 Department of Global HIV, Hepatitis and Sexually Transmitted Infections Programmes, World Health Organization, Geneva, Switzerland

**Keywords:** Sexually transmitted infection, Syphilis, Gonorrhoea, Chlamydia, Lymphogranuloma venereum, Europe, Central Asia

## Abstract

**Background:**

Sexually transmitted infections (STIs) continue to be a significant public health challenge and have an impact on sexual and reproductive health. European Union and European Economic Area (EU/EEA) countries report data annually on the number of cases of syphilis, gonorrhoea, chlamydia, and lymphogranuloma venereum (LGV) to the European Centre for Disease Prevention and Control. No similar system exists for the non-EU/EEA countries in the WHO European Region, and therefore, the sub-regional overview was unclear due to the limited data availability and inconsistency.

**Methods:**

We conducted a survey among 24 WHO member states in the WHO European Region that did not belong to the EU/EEA as of June 2024. The survey collected data on STI surveillance systems and numbers of reported cases of syphilis, gonorrhoea, chlamydia and LGV from 2021 to 2023.

**Results:**

Twenty-one completed the survey, of which 19 (79%) agreed to publish their data. All 19 countries reported surveillance activities for syphilis, 18 for gonorrhoea and chlamydia, and 8 for LGV. The comprehensiveness and coverage of surveillance systems varied between countries and infections. Total cases reported increased from 13,553 to 17,113 (notification rate per 100,000 population 5.4 to 6.9) for syphilis and 12,001 to 13,067 (4.2 to 5.0) for gonorrhoea between 2021 and 2023, while chlamydia cases decreased from 32,556 to 27,802 (13.3 to 11.2). Only one to two LGV cases were reported per year. Various challenges were identified in antimicrobial-resistant gonorrhoea surveillance.

**Conclusions:**

In non-EU/EEA countries, STI cases are likely to be underreported due to incomplete surveillance systems and other factors. Given the increase in the number of reported cases of syphilis and gonorrhoea between 2021 and 2023 and the significant public health consequences of untreated STIs, an accelerated joint effort to strengthen the surveillance systems of the non-EU/EEA countries is warranted to move towards ending epidemics of STIs in the WHO European Region by 2030.

**Supplementary Information:**

The online version contains supplementary material available at 10.1186/s12889-025-22630-6.

## Background

Sexually transmitted infections (STIs) continue to be a significant public health challenge and could have a direct impact on sexual and reproductive health. When left untreated, STIs can lead to long-term irreversible and potentially fatal outcomes, including chronic pelvic pain, cancers, ectopic pregnancies, infertility, adverse pregnancy outcomes, neonatal death, and congenital abnormalities [1]. Some STIs can also increase the risk of human immunodeficiency virus (HIV) acquisition [2]. Despite its significant consequences, STIs often do not cause symptoms or may have long asymptomatic periods, which can result in unknowing transmission during sexual intercourse or during pregnancy [1].

In 2020, there were cumulatively 374 million new cases of the four curable STIs: syphilis, gonorrhoea, chlamydia, and trichomoniasis in adults aged 15–49 years [3]. World Health Organization (WHO) published strategic frameworks to end STIs as public health concerns by 2030 [1, 4]. A 90% reduction in incidence of syphilis and gonorrhoea is required to achieve the 2030 targets [1, 4]. On the other hand, a WHO progress report published in 2024 showed the challenging reality of the control of STIs; the estimated number of new syphilis cases among adults aged 15–49 years increased by over one million in 2022, reaching eight million worldwide, and gonorrhoea case notification rates in selected high-income countries showed an increasing trend [5].

In the WHO European region, the European Centre for Disease Prevention and Control (ECDC) routinely collects data from 30 member states of the European Union and European Economic Area (EU/EEA) and publishes annual reports for syphilis [6], gonorrhoea [7], chlamydia [8], and lymphogranuloma venereum (LGV) [9]. Their latest reports, which covered up to 2023, revealed an increasing trend in reported cases of these diseases, which has raised significant concerns [10]. On the other hand, data from non-EU/EEA countries in the WHO European Region are much more limited, and the latest available data is from a WHO survey of non-EU/ EEA member states covering 2015 to 2019, in which 15 member states responded, and the total number of reported cases syphilis, gonorrhoea and chlamydia fell between 2015 and 2019 [11]. A recent review of STI surveillance records in 53 countries within the WHO European Region in 2023 found limited availability and inconsistency in STI data for non-EU/EEA countries [12].

At the same time, an increasing trend in drug-resistant gonorrhoea has been reported in the EU/EEA [13] and worldwide [14]. However, only six non-EU/EEA countries in the WHO European Region have ever reported to the WHO Gonococcal AMR Surveillance Programme (WHO-GASP) as of 2018 [14]. A previous systematic review by Medland et al. covering 2012 to 2020 identified only a few countries in non-EU/EEA having national surveillance systems for monitoring antimicrobial-resistant gonorrhoea [15].

To address these gaps, the WHO Regional Office for Europe conducted an STI survey sent to 24 non-EU/EEA member states in 2024. This study aimed to collect data on national surveillance systems for STIs, including surveillance for drug-resistant gonorrhoea, reported cases of STIs in adults, and data on prevalence studies. In addition, the survey collected information on congenital syphilis and programmatic indicators linked to the elimination of mother-to-child transmission, which is discussed in a separate paper.

## Methods

### Data collection process

The WHO Regional Office for Europe distributed the survey “WHO Annual Reporting Form on Sexually Transmitted Infections for the period January-December 2021–2023” (see Additional File 1) to the 24 non-EU/EEA WHO member states through official correspondence in June 2024. The countries included in the survey were: Albania, Andorra, Armenia, Azerbaijan, Belarus, Bosnia and Herzegovina, Georgia, Israel, Kazakhstan, Kyrgyzstan, Monaco, Montenegro, North Macedonia, Republic of Moldova, Russian Federation, San Marino, Serbia, Switzerland, Tajikistan, Türkiye, Turkmenistan, Ukraine, United Kingdom, and Uzbekistan (in alphabetical order). National focal point persons were selected by country representatives and asked to complete the survey in English or Russian. The survey collected data on surveillance systems and the number of reported cases of syphilis, gonorrhoea, chlamydia, and LGV. For the reported case data, the countries reported based on their own case definitions.

### Data analysis

#### Surveillance systems

We collected data on national surveillance systems: type of data collection system (universal or sentinel), data sources (public sectors only or both public and private sectors). Also, to evaluate the completeness of the surveillance, countries were asked to estimate reporting coverage [(Number of reported cases/Number of actual cases) *100 (%)] of each infection (< 25%, 26–50%, 51–75%, and 76–100%). In addition, countries were asked whether they have national surveillance systems for monitoring antimicrobial-resistant gonorrhoea (Neisseria gonorrhoeae) and also about the challenges to participating in WHO-GASP.

#### Number of reported cases of syphilis, gonorrhoea, chlamydia and LGV

Based on the number of reported cases of syphilis, gonorrhoea, chlamydia and LGV, notification rates per 100,000 population in each year from 2021 to 2023 were calculated. Notification rates were calculated by dividing the number of reported cases (numerator) by the population of the respective reporting year (denominator) and multiplying by 100,000. For countries using a sentinel surveillance system, numbers of cases were not included in the calculation of national or overall rates, but they were included in the total number of reported cases. Denominators only included the population of countries which provided data for the respective years. Notification rates by gender were also calculated by dividing the number of reported cases in male or female (numerator) by the male or female population of the respective reporting year (denominator) and multiplying by 100,000.

The change in the notification rate of syphilis, gonorrhoea and chlamydia was examined using data from countries that consistently reported the number of cases for three years with available gender disaggregation. Male-to-female ratios were also calculated for syphilis, gonorrhoea, and chlamydia using number of reported cases in 2023. Only the countries which provided gender-disaggregated data were included. The population data was extracted for the years 2021, 2022 and 2023 from the United Nations World Population Prospects [16].

### Ethical statement

Ethical approval was unnecessary for this study because data were collected as part of surveillance activities, and no individual-level data were obtained.

## Results

As of January 2025, 21 member states out of 24 completed the survey (response rate 88%), of which 19 (79%) agreed to publish the data. These countries are: Albania, Andorra, Armenia, Azerbaijan, Belarus, Georgia, Israel, Kyrgyzstan, Monaco, Montenegro, North Macedonia, Republic of Moldova, San Marino, Serbia, Switzerland, Tajikistan, Türkiye, Ukraine, and Uzbekistan (in alphabetical order).

### Surveillance systems

The reported details of surveillance systems for each country are shown in Table 1. All 19 countries reported surveillance activities for syphilis, 18 for gonorrhoea and chlamydia, and 8 for LGV. For the type of data collection system, 15 reported universal surveillance for syphilis, 13 for gonorrhoea and 13 for chlamydia. For syphilis, 11 countries (58%) reported having universal surveillance that covered both the public and private sectors, and 13 countries (68%) estimated coverage was between 76–100%. For gonorrhoea and chlamydia, nine countries (50%) reported having universal surveillance that covered both the public and private sectors, and 10 countries for gonorrhoea (56%) and nine (50%) for chlamydia estimated coverage was between 76–100%. Regarding LGV, of the eight countries that reported having an LGV-specific surveillance system, six used universal surveillance system and four collected data from both the public and private sectors.

**Table 1:** Surveillance systems in 19 non-EU/EEA member states in the WHO European Region

Country	**Syphilis**				**Gonorrhoea**			**Chlamydia**			**LGV**		
	**Universal or Sentinel**	**Data source**		**Coverage**	**Universal or Sentinel**	**Data source**	**Coverage**	**Universal or Sentinel**	**Data source**	**Coverage**	**Universal or Sentinel**	**Data source**	**Coverage**
Albania	U	Both		76–100	No surveillance			No surveillance			No surveillance		
Andorra	U		Both	76–100	U	Both	76–100	U	Both	76–100	U	Both	76–100
Armenia	U	Both		76–100	U	Both	< 25	U	Both	< 25	No surveillance		
Azerbaijan	U	Public		76–100	U	Public	76–100	U	Public	76–100	U	Public	NDR
Belarus	U	Both		76–100	S	Public	76–100	S	Public	76–100	U	Both	NDR
Georgia	U	Both		76–100	U	Both	76–100	U	Both	76–100	No surveillance		
Israel	U	Public		NDR	U	Public	NDR	U	Public	NDR	No surveillance		
Kyrgyzstan	NDR	Both		76–100	NDR	Both	76–100	NDR	Both	NDR	No surveillance		
Monaco	S	Public		< 25	S	Public	< 25	S	Public	< 25	S	Public	< 25
Montenegro	U	Both		< 25	U	Both	< 25	U	Both	< 25	No surveillance		
North Macedonia	U	Both		NDR	U	Both	NDR	U	Both	NDR	U	Both	NDR
Republic of Moldova	U	Both		76–100	U	Both	76–100	U	Both	76–100	No surveillance		
San Marino	U	Public		76–100	U	Public	76–100	U	Public	76–100	U	Public	NDR
Serbia	U	Both		76–100	U	Both	26–50	U	Both	26–50	U	Both	NDR
Switzerland	U	Both		76–100	U	Both	76–100	U	Both	76–100	Part of chlamydia surveillance		
Tajikistan	NDR	NDR		NDR	NDR	NDR	NDR	NDR	NDR	NDR	NDR	NDR	NDR
Türkiye	U	Both		76–100	U	Both	76–100	U	Both	76–100	No surveillance		
Ukraine	U	Public		NDR	U	Public	NDR	U	Public	NDR	No surveillance		
Uzbekistan	S	Public		76–100	S	Public	76–100	S	Public	76–100	S	Public	76–100

N*DR* no data reported

Table 2 summarizes the data on surveillance for antimicrobial-resistant gonorrhoea. Four countries, Georgia, Israel, North Macedonia and Switzerland, reported having national gonococcal AMR monitoring systems. Table 2 also shows the countries that reported to the WHO GASP. Among four countries with a national monitoring system, three did not report to WHO GASP. North Macedonia mentioned a lack of information as a reason for not reporting. In addition, five countries that did not collect data on AMR provided information on the challenges to participating in WHO GASP. These included a lack of laboratory capacity, funding, infrastructure, technical expertise, a coordinating body, and a single electronic data collection system.

**Table 2:** Drug-resistant gonorrhoea surveillance in 19 non-EU/EEA member states in the WHO European Region

Country	National Surveillance	Reporting to WHO-GASP	Challenges mentioned to participating in WHO-GASP
Albania	No	No	N/A
Andorra	No	No	N/A
Armenia	No	No	Lack of national AMR surveillance programs, Resource limitations, Technical expertise and capacity gaps, Limited coordination mechanisms
Azerbaijan	No	No	N/A
Belarus	No	No^a^	N/A
Georgia	Yes	No	N/A
Israel	Yes	No^a^	N/A
Kyrgyzstan	No	No^a^	N/A
Monaco	No	No	N/A
Montenegro	No	No	No referent laboratory, Low testing rates, Limited number of isolates
North Macedonia	Yes	No	No information on how to report to WHO GASP
Republic of Moldova	No	No	Lack of infrastructure
San Marino	No	Unknown	N/A
Serbia	No	No	No national program, No national reference laboratory
Switzerland	Yes	Yes	N/A
Tajikistan	No	Unknown	N/A
Türkiye	No	Unknown	N/A
Ukraine	No	No^a^	Lack of a single electronic data collection system, Insufficient provision of laboratory reagents, Lack of requirements
Uzbekistan	No	Unknown	N/A

*N/A* not available

^a^These countries have reported to WHO GASP at least once by 2020 according to GHO data: https://www.who.int/data/gho/data/themes/topics/indicator-groups/indicator-group-details/GHO/number-of-isolates-tested-(who-gasp-amr)

### Number of reported syphilis, gonorrhoea, chlamydia and LGV cases

Nineteen countries reported data on syphilis, 18 on gonorrhoea, and 17 on chlamydia for all three years. The number of cases and notification rates per 100,000 population for syphilis, gonorrhoea and chlamydia are shown in Table 3. In 2021, there were 13,553 reported syphilis cases from the 19 non-EU/EEA member states, which is equivalent to a notification rate of 5.4 per 100,000. The number of cases and rate increased to 15,960 (6.4 per 100,000) in 2022 and 17,113 (6.9 per 100,000) in 2023. A similar change was also observed for gonorrhoea; the number of cases increased from 12,001 in 2021 (4.2 per 100,000) to 12,544 in 2022 (4.5 per 100,000) and then to 13,067 (5.0 per 100,000) in 2023. Unlike syphilis and gonorrhoea, reported cases of chlamydia fell between 2021 and 2023; in 2021, there were 32,556 reported cases (13.3 per 100,000), and in 2023, 27,802 (11.2 per 100,000). To assess the change in the longer term, we compared the average number of cases from 2021 to 2023 with the latest available data from our previous STI survey between 2015 and 2019 [11] (see Additional File 2). Syphilis cases increased in eight out of 12 countries with available data. Gonorrhoea and chlamydia decreased in nine out of 11 and nine out of 10 countries, respectively. For LGV, only eight countries in 2023 and seven countries in 2021 and 2022 reported the number of cases, of which two countries have a sentinel surveillance system. The reporting countries were Andorra (starting from 2023), Azerbaijan, Belarus, North Macedonia, Monaco, San Marino, Serbia and Uzbekistan. Among these eight countries, six reported zero cases for all reporting years, and there was one reported case in 2021 and 2023 (both from Monaco), and two cases were reported (Monaco and Uzbekistan) in 2022.

**Table 3:** Total number of reported cases and notification rates of syphilis, gonorrhoea and chlamydia with national data from 19 non-EU/EEA member states, the WHO European Region, 2021–2023

**Country**	**Syphilis**						**Gonorrhoea**						**Chlamydia**					
	**2021**		**2022**		**2023**		**2021**		**2022**		**2023**		**2021**		**2022**		**2023**	
	**N**	**Rate**	**N**	**Rate**	**N**	**Rate**	**N**	**Rate**	**N**	**Rate**	**N**	**Rate**	**N**	**Rate**	**N**	**Rate**	**N**	**Rate**
**Albania**	47	1.6	18	0.6	111	3.9	NDR		NDR		NDR		NDR		NDR		NDR	
**Andorra**	0	0.0	3	3.8	5	6.2	2	2.6	2	2.5	11	14.1	NDR		NDR		4	4.9
**Armenia**	439	15.3	604	21.0	710	25.5	271	9.4	241	8.4	193	6.7	916	31.9	492	17.1	604	20.5
**Azerbaijan**	837	8.2	1,127	10.9	1,130	10.9	252	2.5	228	2.2	327	3.2	610	6.0	395	3.8	219	2.1
**Belarus**	975	10.5	1129	12.3	1,101	11.5	710	NRC	956	NRC	899	NRC	3,081	NRC	2,615	NRC	2,610	NRC
**Georgia**	1,096	28.9	1,189	31.3	1,263	33.8	498	13.1	478	12.6	497	13.1	1,111	29.3	927	24.4	1,039	27.3
**Israel**	632	7.1	696	7.6	793	8.8	846	9.5	639	7.0	664	7.4	1,501	16.8	907	10.0	906	9.8
**Kyrgyzstan**	274	4.0	348	5.0	479	7.2	146	2.1	209	3.0	178	2.6	1,243	18.2	1,041	15.0	907	12.8
**Monaco**	4	NRC	6	NRC	11	NRC	1	NRC	1	NRC	1	NRC	1	NRC	1	NRC	1	NRC
**Montenegro**	5	0.8	4	0.7	6	1.0	0	0.0	0	0.0	7	1.2	15	2.5	10	1.6	1	0.2
**North Macedonia**	5	0.3	22	1.2	60	2.9	0	0.0	0	0.0	8	0.4	34	1.8	31	1.7	26	1.4
**Republic of Moldova**	1,269	42.0	1,324	43.6	1,142	34.9	544	18.0	431	14.2	285	9.4	2,066	68.3	1,954	64.3	1,544	50.3
**San Marino**	18	51.4	11	32.4	16	53.3	1	2.9	5	14.7	1	2.9	4	11.4	4	11.8	6	17.6
**Serbia**	176	2.6	215	3.2	211	2.9	106	1.6	79	1.2	121	1.8	319	4.7	358	5.3	458	6.8
**Switzerland**	729	8.4	844	9.6	810	9.3	4,079	46.8	5,122	58.3	6,101	70.1	12,335*	141.7	13,120*	149.2	12,780*	144.1
**Tajikistan**	266	2.7	333	3.3	324	3.3	136	1.4	106	1.0	69	0.7	124	1.2	161	1.6	136	1.3
**Türkiye**	2,785	3.2	3,542	4.1	3,646	4.3	57	0.1	136	0.2	84	0.1	62	0.1	31	0.0	32	0.0
**Ukraine**	1,540	3.5	1,581	3.9	2,053	5.2	1,296	2.9	952	2.3	872	2.0	5,582	12.6	3,522	8.6	3,389	9.0
**Uzbekistan**	2,456	NRC	2,964	NRC	3,242	NRC	3,056	NRC	2,959	NRC	2,749	NRC	3,552	NRC	3,422	NRC	3,140	NRC
**Total**	13,553	5.4	15,960	6.4	17,113	6.9	12,001	4.2	12,544	4.5	13,067	5.0	32,556	13.3	28,991	11.5	27,802	11.2

*NDR* no data reported, *NRC* no rate calculated (because the numbers were based on sentinel surveillance)

^*^including LGV

Disaggregated data by gender was available consistently for 18 member states for syphilis and 17 for gonorrhoea and chlamydia.

**Fig. 1:**
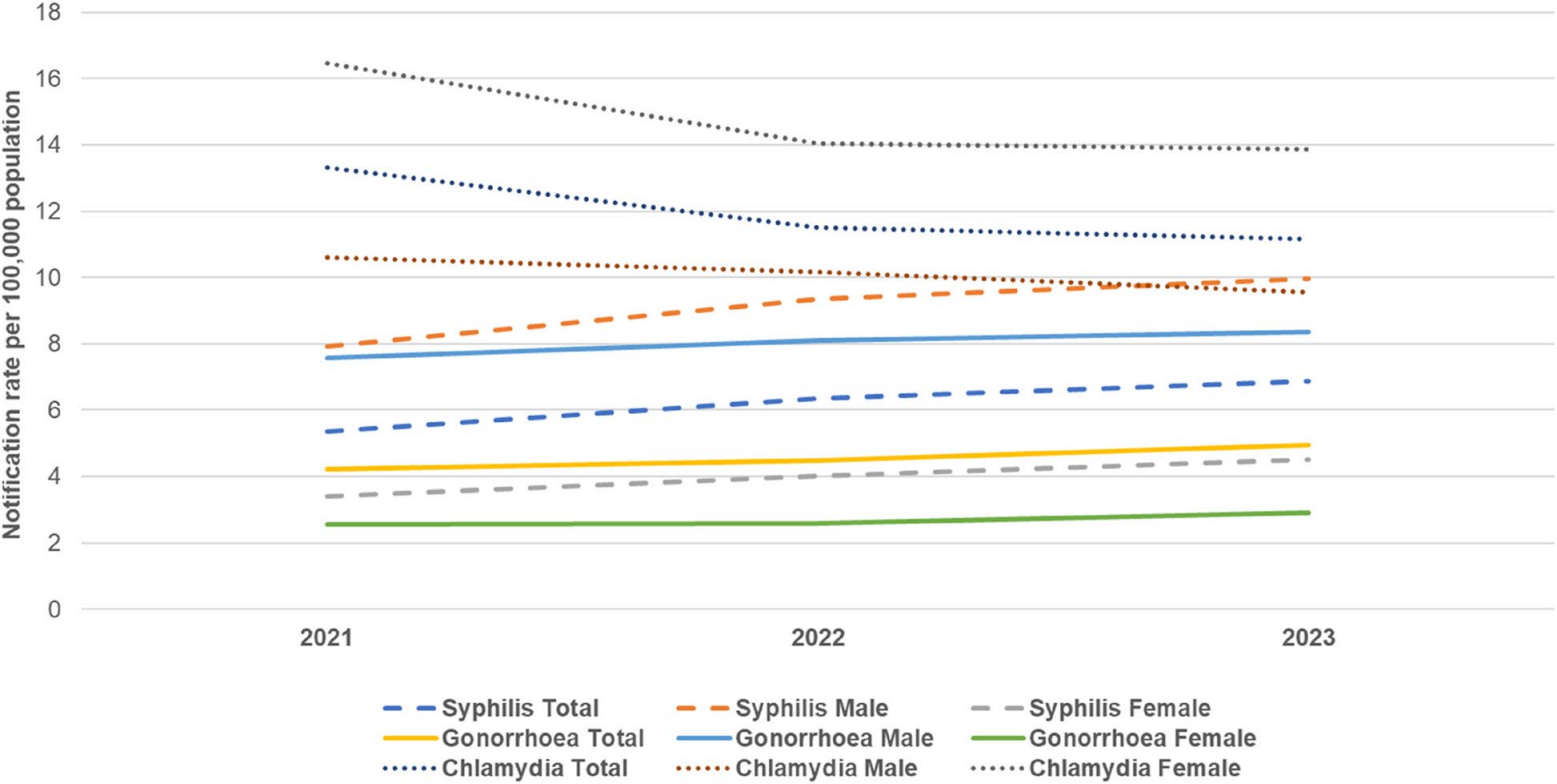
Notification rates per 100,000 population for Syphilis, Gonorrhoea and Chlamydia, 2021–2023 Notification rates per 100,000 population for syphilis, gonorrhoea and chlamydia in total and by gender from 2021 to 2023. Andorra was not included in the calculation of notification rates by gender as gender disaggregation was not available consistently

Figure 1 shows notification rates per 100,000 population for syphilis, gonorrhoea and chlamydia in total and by gender from 2021 to 2023. For syphilis, the overall notification rate increased in both men and women from 2021 to 2023: 7.9 to 10.0 (+ 27%) for men and 3.4 to 4.5 (+ 32%) for women. Similarly, for gonorrhoea, rates increased in the same period by 10% (7.6 to 8.3 per 100,000) in men and by 13% (2.6 to 2.9 per 100,000) in women. The decrease in chlamydia was observed among both genders, but women (notification rate of 16.5 in 2021 to 13.9 in 2023, 16% reduction) contributed more to the decline than men (10.6 in 2021 to 9.6 in 2023, 10% reduction) (Fig. 1).

**Fig. 2:**
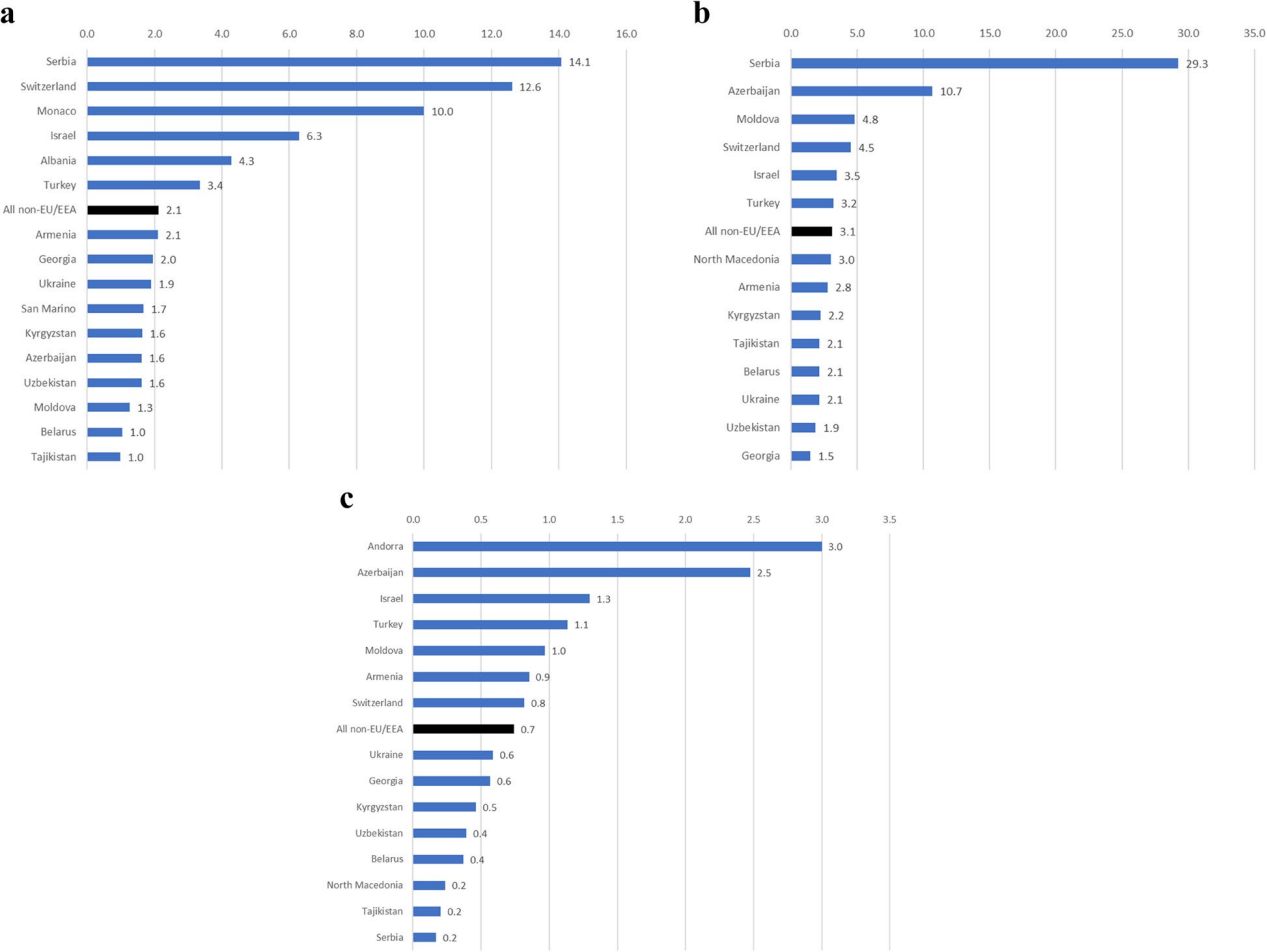
Male-to-female ratio by infection and country, 2023. **a** syphilis, **b** gonorrhoea, **c** chlamydia National and all non-EU/EEA male-to-female ratios for reported cases in 2023. **a** Ratios for Andorra, Montenegro and North Macedonia are not available as these countries reported zero female cases. **b** Ratios are not available for Andorra, Monaco and Montenegro (zero female cases) and San Marino (zero male cases). Gender-disaggregated data was not available for Albania. **c** Male-to-female ratio of chlamydia cases by country, 2023. National and all non-EU/EEA male-to-female ratios for reported chlamydia cases in 2023. Ratios are not available for Monaco (0 female cases) and Montenegro and San Marino (0 male cases). Gender-disaggregated data was not available for Albania

The national and overall male-to-female ratio for syphilis, gonorrhoea and chlamydia are shown in Fig. 2 (a, b, c). For syphilis and gonorrhoea, the male-to-female ratio in all countries was 1.0 or greater. For syphilis, the overall male-to-female ratio was 2.1 in 2023 [range 1.0 in Tajikistan to 14.1 in Serbia]. For gonorrhoea, the overall ratio was 3.1 [range 1.5 (Georgia) – 29.3 (Serbia)]. For chlamydia, the ratio was less than 1.0 in 11 of 15 countries (an overall ratio of 0.7, ranging from 0.2 in Serbia to 3.0 in Andorra).

While all reporting countries have sex-disaggregated data for at least one infection as of 2023, only five countries had available modes of transmission data. Regarding age disaggregation, 15 countries provided the number of cases for pre-defined age groups used by ECDC: 14, 15–19, 20–24, 24–34, 35–44, 45 and over, and four were unable to report the numbers based on this age grouping and used different grouping.

## Discussion

To our knowledge, this is the first study which revealed an increase in STIs in non-EU/EEA. Before this study, the sub-regional overview was unclear due to the limited data availability and inconsistency. This study employed a uniform data collection method, achieved a high response rate and revealed an increase in syphilis and gonorrhoea among the non-EU/EEA sub-region.

Syphilis showed an increase with a notification rate of 5.4 in 2021 to 6.9 in 2023, which was similar to the reported rates for EU/EEA (6.4 in 2021 and 10.0 in 2023) [17]. While Gonorrhoea cases also increased, larger gaps were observed in notification rate; the rates for non-EU/EEA and EU/EEA were 4.2 and 11.8 in 2021 and 5.0 and 25.0 in 2023, respectively [17]. Chlamydia showed a constant decline, which is not consistent with reports from the EU/EEA, and there were significant gaps in rates between non-EU/EEA and EU/EEA; the rates for non-EU/EEA and EU/EEA were 13.3 and 76.2 in 2021, 11.5 and 89.0 in 2022, and 11.2 and 70.4 in 2023, respectively [17]. There was a correlation between the completeness of surveillance systems in the non-EU/EEA countries and gaps in the notification rate between non-EU/EEA and EU/EEA; the gap is narrower in diseases with more comprehensive surveillance systems, such as syphilis. It is unclear to what extent these gaps between EU/EEA and non-EU/EEA countries reflected a true difference in incidence or resulted from gaps in the completeness of reporting. In a previous narrative review on bacterial STIs in non-EU/EEA, the rates for Syphilis (11.3 per 100,000 population in 2021), Gonorrhoea (6.27 per 100,000) and Chlamydia (23.76 per 1000,000) were higher than our results [12]. This might be because of the difference in data sources; our study used national data collected uniformly in the same timeframe and included data from the countries that the previous study did not include. Comparison with data from our previous survey suggested that an increase in syphilis and a decrease in chlamydia can also be observed in the long term. However, because of the limited availability and inconsistency of the previous data, further analyses were difficult. To assess the long-term trend of STIs in the non-EU/EEA, it is important to continue the coordinated data collection process on a regular basis.

There was not adequate data for LGV as most countries do not have national surveillance systems. LGV type of chlamydia has many specific characteristics: epidemiologically concentrated among men who have sex with men, more invasive and systemic infection, and if not treated early, leading to various chronic conditions [18]. Therefore, LGV needs separate surveillance systems from general chlamydia. There is a clear need in non-EU/EEA to develop surveillance systems to monitor LGV cases. Considering that LGV cases are substantially underdiagnosed across Europe [19], in addition to strengthening surveillance, measures such as raising awareness, training for healthcare workers, and improving access to testing should be combined in an integrated approach.

At the same time, this study sheds light on the huge challenges of STI surveillance in this sub-region. While most countries had surveillance activities for syphilis, gonorrhoea and chlamydia, comprehensiveness and coverage of the systems were suboptimal. Along with a low number of countries with LGV-specific surveillance, these gaps in the surveillance systems suggest a high probability of underreporting and a strong need for strengthening STI surveillance in the non-EU/EEA countries. The existing gaps in STI surveillance could hinder effective analysis, which can inform the policy. In this study, we could only collect aggregated data as most countries do not have a case-based data collection system, which makes further analyses on reported cases difficult. Due to the different age groups among the reporting countries, we could not analyse the notification rate by age group. For example, in the EU/EEA, a sharp rise in gonorrhoea notification rate in young people, particularly women aged 20–24 years, was observed in 2022–2023 [20]. However, we could not examine if there was a similar change in non-EU/EEA due to a lack of age disaggregation. Similarly, due to the significantly limited data availability, it was not possible to analyse the mode of transmission. In the context of the scale-up of pre-exposure prophylaxis (PrEP) for HIV in non-EU/EEA [21], it is particularly important to monitor the incidence of STIs among key populations, such as men who have sex with men, as PrEP does not protect against other STIs than HIV and periodic testing for syphilis, gonorrhoea and chlamydia is strongly recommended by WHO [22]. Age and mode of transmission disaggregation are indispensable in revealing the accurate epidemics of STI transmissions and identifying specific populations who are most at risk for STIs to inform the policy about effective measures.

Another issue in surveillance systems was limited monitoring for antimicrobial-resistant gonorrhoea; a dedicated surveillance system has not been established in most countries. One country mentioned the usefulness of established collaboration with regional or international bodies. Considering the public health impact due to the possible spread of antimicrobial-resistant gonorrhoea, including multi-drug resistant gonorrhoea [23], this study showed the vulnerability of non-EU/EEA countries. The challenges countries mentioned mainly resulted from a lack of political commitment and resources. To rapidly detect antimicrobial-resistant gonorrhoea and prevent its further spread, a regional and global collaborative approach to support developing surveillance at the national level should be considered.

Taken together, non-EU/EEA countries share many common challenges in STI surveillance, and increased domestic and international support and the establishment of a coordinated international STI data reporting system are urgently needed to monitor and ensure that STI resources are used efficiently to control STIs in non-EU/EEA effectively.

The strength of this study is the high number of countries providing data, which enabled us to understand the overall picture of STI epidemics and identify common challenges in STI surveillance systems of this sub-region. At the same time, the data in this study should be interpreted in the light of several limitations. Firstly, a high heterogeneity between reporting countries in not only surveillance systems but also testing policies, access to STI testing, diagnostic techniques and reporting practices should be taken into account. Also, we did not define case definitions for the infections, and the countries used their own definitions. Possible heterogeneity in how the reported cases are defined should be considered. Second, this study is based on the responses from the countries, and no data in the collection form does not necessarily mean that there is no such data. Finally, considering the sub-regional context of high levels of stigma and discrimination [24, 25] and suboptimal access to and quality of testing [26] along with the above-mentioned suboptimal surveillance systems, it is highly likely that the number of STI cases is underreported, and the real scale of the STI epidemic in non-EU/EEA can be bigger than reported.

## Conclusions

In non-EU/EEA countries in the WHO European Region, the number of reported cases of syphilis and gonorrhoea increased between 2021 and 2023. At the same time, this study sheds light on the enormous challenges of STI surveillance in this subregion. Given the significant public health consequences of untreated STIs, an accelerated joint effort to strengthen the surveillance systems of the non-EU/ EEA countries is warranted to move towards ending epidemics of STIs in the WHO European Region by 2030.

## Supplementary Information

Supplementary Material 1.

## Data Availability

The results of the surveys are available from the corresponding author upon reasonable request.

## Abbreviations

ECDC: European Centre for Disease Prevention and Control
EU/EEA: European Union and European Economic Area
HIV: Human immunodeficiency virus
LGV: Lymphogranuloma venereum
STIs: Sexually transmitted infections
WHO: World Health Organization
WHO-GASP: WHO Gonococcal AMR Surveillance Programme

## References

[1] World Health Organization (WHO). Global health sector strategies on, respectively, HIV, viral hepatitis and sexually transmitted infections for the period 2022–2030. 2022. https://iris.who.int/handle/10665/338901.

[2] Boily MC, et al. Heterosexual risk of HIV-1 infection per sexual act: a systematic review and meta-analysis of observational studies. Lancet Infect Dis. 2009;9(2):118. 10.1016/S1473-3099(09)70021-0. 19179227 10.1016/S1473-3099(09)70021-0 PMC4467783

[3] World Health Organization (WHO). Global progress report on HIV, viral hepatitis and sexually transmitted infections, 2021. 2021.

[4] World Health Organization (WHO) Regional Office for Europe. Regional action plans for ending AIDS and the epidemics of viral hepatitis and sexually transmitted infections 2022–2030. Copenhagen: WHO Regional Office for Europe; 2023. Licence: CC BY-NC-SA 3.0 IGO.

[5] World Health Organization (WHO). Implementing the global health sector strategies on HIV, viral hepatitis and sexually transmitted infections, 2022–2030: report on progress and gaps 2024. 2024.

[6] European Centre for Disease Prevention and Control. Syphilis - annual epidemiological report 2023. 2025. Available: https://www.ecdc.europa.eu/en/publications-data/syphilis-annual-epidemiological-report-2023.

[7] European Centre for Disease Prevention and Control and WHO Regional Office for Europe. Gonorrhoea - annual epidemiological report for 2023. 2025. Available: https://www.ecdc.europa.eu/en/publications-data/gonorrhoea-annual-epidemiological-report-2023.

[8] European Centre for Disease Prevention and Control. Chlamydia - annual epidemiological report for 2023. 2025. Available: https://www.ecdc.europa.eu/en/publications-data/chlamydia-annual-epidemiological-report-2023.

[9] European Centre for Disease Prevention and Control. Lymphogranuloma venereum - annual epidemiological report for 2023. 2025. Available: https://www.ecdc.europa.eu/en/publications-data/lymphogranuloma-venereum-annual-epidemiological-report-2023.

[10] European Centre for Disease Prevention and Control. STI cases continue to rise across Europe. https://www.ecdc.europa.eu/en/news-events/sti-cases-continue-rise-across-europe. Accessed 11 Feb 2025.

[11] Barbaric J, et al. Surveillance and epidemiology of syphilis, gonorrhoea and chlamydia in the non-European Union countries of the World Health Organization European Region, 2015 to 2020. Eurosurveillance. 2022;27(8):1–11. 10.2807/1560-7917.ES.2022.27.8.2100197. 10.2807/1560-7917.ES.2022.27.8.2100197 PMC8874864 35209970

[12] Mitjà O, et al. Epidemiology and determinants of reemerging bacterial sexually transmitted infections (STIs) and emerging STIs in Europe. Lancet Reg Heal Eur. 2023;34:1–17. 10.1016/j.lanepe.2023.100742. 10.1016/j.lanepe.2023.100742 PMC10625005 37927427

[13] European Centre for Disease Prevention and Control. Gonococcal antimicrobial susceptibility surveillance in the European Union/European Economic Area - summary of results for 2020. 2024. Available: https://www.ecdc.europa.eu/en/publications-data/gonococcal-antimicrobial-susceptibility-surveillance-2020.

[14] World Health Organization (WHO). Global Health Observatory: gonococcal antimicrobial susceptibilities: number of isolates tested (WHO-GASP). 2024. https://www.who.int/data/gho/data/themes/topics/indicator-groups/indicator-group-details/GHO/number-of-isolates-tested-(who-gasp-amr). Accessed 10 Feb 2025.

[15] NA Medland et al. Surveillance systems to monitor antimicrobial resistance in Neisseria gonorrhoeae: a global, systematic review, 1 January 2012 to 27 September 2020. Eurosurveillance. 2022;27(18). 10.2807/1560-7917.ES.2022.27.18.2100917. 10.2807/1560-7917.ES.2022.27.18.2100917 PMC9074396 35514308

[16] United Nations Department of Economic and Social Affairs. World population prospects 2024. 2024. https://population.un.org/wpp/. Accessed 01 Oct 2024.

[17] European Centre for Disease Prevention and Control. Surveillance atlas of infectious diseases. https://atlas.ecdc.europa.eu/public/index.aspx. Accessed 24 Jan 2025.

[18] World Health Organization (WHO). Chlamydia Key facts. 2024. https://www.who.int/news-room/fact-sheets/detail/chlamydia.

[19] Cole MJ, et al. Substantial underdiagnosis of lymphogranuloma venereum in men who have sex with men in Europe: preliminary findings from a multicentre surveillance pilot. Sex Transm Infect. 2020;96(2):137–42. 10.1136/sextrans-2019-053972. 31235527 10.1136/sextrans-2019-053972 PMC7035679

[20] Nerlander L, et al. Sharp increase in gonorrhoea notifications among young people, EU/EEA, July 2022 to June 2023. Eurosurveillance. 2024;29(10):1–6. 10.2807/1560-7917.ES.2024.29.10.2400113. 10.2807/1560-7917.ES.2024.29.10.2400113 PMC10986672 38456219

[21] Gokengin D, et al. PrEP Scale-Up and PEP in Central and Eastern Europe: changes in time and the challenges we face with no expected HIV vaccine in the near future. Vaccines. 2023;11(1):3–14. 10.3390/vaccines11010122. 10.3390/vaccines11010122 PMC9867039 36679967

[22] World Health Organization (WHO). Implementation tool for pre-exposure prophylaxis of HIV infection - integrating STI services. 2022. https://www.who.int/publications/i/item/9789240097230. Accessed 14 Nov 2024.

[23] Jensen JS, Unemo M. Antimicrobial treatment and resistance in sexually transmitted bacterial infections. Nat Rev Microbiol. 2024;22(7):435–50. 10.1038/s41579-024-01023-3. 38509173 10.1038/s41579-024-01023-3

[24] Garcia PJ, Miranda AE, Gupta S, Garland SM, Escobar ME, Fortenberry JD. The role of sexually transmitted infections (STI) prevention and control programs in reducing gender, sexual and STI-related stigma. EClinicalMedicine. 2021;33:100764. 10.1016/j.eclinm.2021.100764. 33718850 10.1016/j.eclinm.2021.100764 PMC7921479

[25] Uusküla A, Kangur K, McNutt LA. Barriers to effective STI screening in a post-Soviet society: results from a qualitative study. Sex Transm Infect. 2006;82(4):323–6. 10.1136/sti.2005.019000. 16877585 10.1136/sti.2005.019000 PMC2564721

[26] Boiko I, Krynytska I, Kohut I, Bezkorovaina H, Stepanenko V. Diagnostics of gonococcal infection in Ukraine: current challenges in resource-constrained settings. Eurasian J Med. 2021;53(3):180. 10.5152/EURASIANJMED.2021.20043. 35110093 10.5152/eurasianjmed.2021.20043 PMC9879215

