# Supplementary material for: Sexually transmitted infections in the non-European Union and European Economic Area of the World Health Organization European Region 2021–2023
Source: BMC Public Health. 2025 Apr 25;25:1545. doi: 10.1186/s12889-025-22630-6 (PMC12023690; doi:10.1186/s12889-025-22630-6)
Supplement: Supplementary file 1 — Supplementary Material 1. [file 12889_2025_22630_MOESM1_ESM.docx]

# Additional File 1. WHO Annual Reporting Form on Sexually Transmitted Infections for the period January-December, 2021-2023

**1-3. Registered cases of sexually transmitted infections in 2021, 2022, 2023 (one table for each year)**

| **Disease** | **Gender** | **Female** | | | **Male** | | | | **Unknown** |
| --- | --- | --- | --- | --- | --- | --- | --- | --- | --- |
|  |  | transmission route | | | transmission route | | | | Total |
|  | By age group | Heterosexual | Other | Unknown | MSM | Heterosexual | Other | Unknown |  |
| **Syphilis**  **(Primary, Secondary, Early latent)** | 0-14 |  |  |  |  |  |  |  |  |
|  | 15-19 |  |  |  |  |  |  |  |  |
|  | 20-24 |  |  |  |  |  |  |  |  |
|  | 25-34 |  |  |  |  |  |  |  |  |
|  | 35-44 |  |  |  |  |  |  |  |  |
|  | 45+ |  |  |  |  |  |  |  |  |
|  | unknown |  |  |  |  |  |  |  |  |
| **Gonorrhoea** | 0-14 |  |  |  |  |  |  |  |  |
|  | 15-19 |  |  |  |  |  |  |  |  |
|  | 20-24 |  |  |  |  |  |  |  |  |
|  | 25-34 |  |  |  |  |  |  |  |  |
|  | 35-44 |  |  |  |  |  |  |  |  |
|  | 45+ |  |  |  |  |  |  |  |  |
|  | unknown |  |  |  |  |  |  |  |  |
| **Chlamydia (excluding Lymphogranuloma venereum)** | 0-14 |  |  |  |  |  |  |  |  |
|  | 15-19 |  |  |  |  |  |  |  |  |
|  | 20-24 |  |  |  |  |  |  |  |  |
|  | 25-34 |  |  |  |  |  |  |  |  |
|  | 35-44 |  |  |  |  |  |  |  |  |
|  | 45+ |  |  |  |  |  |  |  |  |
|  | unknown |  |  |  |  |  |  |  |  |
| **Lymphogranuloma venereum (LGV)** | 0-14 |  |  |  |  |  |  |  |  |
|  | 15-19 |  |  |  |  |  |  |  |  |
|  | 20-24 |  |  |  |  |  |  |  |  |
|  | 25-34 |  |  |  |  |  |  |  |  |
|  | 35-44 |  |  |  |  |  |  |  |  |
|  | 45+ |  |  |  |  |  |  |  |  |
|  | unknown |  |  |  |  |  |  |  |  |

**4. Congenital syphilis**

**4.1. Please indicate the total number of congenital syphilis cases reported in 2021-2023 and whether stillbirths are included or not.**

| **Year** | **The total number of cases** | **Stillbirths included?** |
| --- | --- | --- |
| **2021** |  | **( Yes No Unknown )** |
| **2022** |  | **( Yes No Unknown )** |
| **2023** |  | **( Yes No Unknown )** |

**4.2. Please indicate the case definition of congenital syphilis if it is specified in reporting.**

**4.3. Please indicate the percentage and number of pregnant women attending antenatal care who were screened for syphilis, the percentage that tested positive, and the percentage that was treated.**

| **Year** | **Number tested** | **Percentage tested** | **Number positive** | **Percentage positive** | **Number treated** | **Percentage treated** |
| --- | --- | --- | --- | --- | --- | --- |
| **2021** |  | **%** |  | **%** |  | **%** |
| **2022** |  | **%** |  | **%** |  | **%** |
| **2023** |  | **%** |  | **%** |  | **%** |

**5. Priority populations**

**5.1 Men who have sex with men**

Is there any ongoing or completed study in the last five years on the prevalence and/or trend of **syphilis** or **gonorrhoea** among **men who have sex with men**? If so, please provide information and key results.

**5.2 Female sex workers**

Is there any ongoing or completed study in the last five years on the prevalence and/or trend of **syphilis** or **gonorrhoea** among **female sex workers**? Please indicate the data and source, if any.

**5.3 Other populations**

Are there any studies on the prevalence and/or trend of syphilis or gonorrhoea among **other specific populations** in the last five years? If so, please provide details on the population and results.

**6. Surveillance system**

**6.1. Please fill in the table below about the surveillance system and data source.**

**Please note that universal reporting implies that all healthcare providers who diagnose STIs are expected to report, while sentinel reporting means that reporting is done only by selected healthcare facilities.*

| ***Disease*** | ***Surveillance system*** | ***Data source*** |
| --- | --- | --- |
| Syphilis  (Primary, Secondary, Early latent) | (universal sentinel) | (Only public sector, Public and private sector) |
| Gonorrhoea | (universal sentinel) | (Only public sector, Public and private sector) |
| Chlamydia  (excluding Lymphogranuloma venereum (LGV)) | (universal sentinel) | (Only public sector, Public and private sector) |
| Lymphogranuloma venereum (LGV) | (universal sentinel) | (Only public sector, Public and private sector) |
| Congenital syphilis | (universal sentinel) | (Only public sector, Public and private sector) |

**6.2. Please choose the estimated reporting coverage* of each disease from below 25%, 26-50%, 51-75%, and 76-100%.**

**(Number of reported cases/Number of actual cases) *100 (%)*

| **Disease** | **Estimated coverage (%)** |
| --- | --- |
| Syphilis (Primary, Secondary, Early latent) | <25, 26-50, 51-75, 76-100 |
| Gonorrhoea | <25, 26-50, 51-75, 76-100 |
| Chlamydia (excluding Lymphogranuloma venereum (LGV)) | <25, 26-50, 51-75, 76-100 |
| Lymphogranuloma venereum (LGV) | <25, 26-50, 51-75, 76-100 |
| Congenital syphilis | <25, 26-50, 51-75, 76-100 |

**6.3.** **Is your country reporting Neisseria gonorrhoea antimicrobial resistance (AMR) to the WHO Gonococcal Antimicrobial Surveillance Programme (GASP)? If not, please describe the challenges to participating.**

**6.4. Is there any surveillance system for AMR surveillance for Neisseria gonorrhoeae in your country? If so, please provide details.**

**Any Additional Comments**

# Additional File 2. The number of reported cases of syphilis, gonorrhoea and chlamydia from the previous STI survey from 2015 to 2019, WHO European Region

|  | **Syphilis** | | | | **Gonorrhoea** | | | | **Chlamydia** | | | |  |
| --- | --- | --- | --- | --- | --- | --- | --- | --- | --- | --- | --- | --- | --- |
| **Country** | 2021-2023 average | **Latest data*** | **Year** | **Rate** | 2021-2023 average | **Latest data*** | **Year** | **Rate** | 2021-2023 average | **Latest data*** | **Year** | **Rate** |  |
|  |  |  |  |  |  |  |  |  |  |  |  |  |  |
| **Albania** | 59 | 68 | 2018 | 2.4 | - | NDR | - | - | - | NDR | - | - |  |
| **Armenia** | 584 | 110 | 2019 | 3.7 | 235 | 291 | 2019 | 9.8 | 671 | 779 | 2019 | 26.3 |  |
| **Azerbaijan** | 1031 | 824 | 2019 | 8.2 | 269 | 306 | 2019 | 3 | 408 | 1910 | 2019 | 19 |  |
| **Belarus** | 1068 | 405 | 2019 | 4.3 | 855 | 767 | 2019 | 8.1 | 2769 | 4095 | 2019 | 43.3 |  |
| **Georgia** | 1183 | 1059 | 2019 | 26.5 | 491 | 738 | 2019 | 18.5 | 1026 | 1559 | 2019 | 39 |  |
| **Kyrgyzstan** | 367 | 371 | 2016 | 6.1 | 178 | 215 | 2016 | 3.5 | 1064 | 1736 | 2015 | 29.1 |  |
| **Montenegro** | 4 | 3 | 2017 | 0.5 | 2 | 4 | 2017 | 0.6 | 9 | 15 | 2017 | 2.4 |  |
| **North Macedonia** | 29 | 8 | 2018 | 0.4 | 3 | 0 | 2018 | 0 | 30 | 95 | 2018 | 4.6 |  |
| **Serbia** | 201 | 206 | 2018 | 2.3 | 102 | 71 | 2018 | 0.8 | 378 | 879 | 2018 | 10 |  |
| **Tajikistan** | 308 | 360 | 2018 | 4 | 104 | 243 | 2018 | 2.7 | 140 | 47 | 2018 | 0.5 |  |
| **Ukraine** | 1725 | 2486 | 2019 | 5.7 | 1040 | 3263 | 2019 | 7.4 | 4164 | 13907 | 2019 | 31.6 |  |
| **Uzbekistan** | 2887 | 2874 | 2019 | 8.7 | 2921 | 3540 | 2019 | 10.7 | - | N/A | - | - |  |

NDR; no data reported, N/A; not available
